# Gene-Environment Interaction Loci Associated with Refractive Error: SCAMPI Analysis

**DOI:** 10.1016/j.xops.2026.101219

**Published:** 2026-05-05

**Authors:** Xi He, Louise Terry, Jeremy A. Guggenheim, UK Biobank Eye and Vision Consortium, Naomi Allen, Tariq Aslam, Denize Atan, Konstantinos Balaskas, Sarah Barman, Jenny Barrett, Paul Bishop, Graeme Black, Tasanee Braithwaite, Roxana Carare, Usha Chakravarthy, Michelle Chan, Sharon Chua, Annegret Dahlmann-Noor, Alexander Day, Parul Desai, Bal Dhillon, Andrew Dick, Alexander Doney, Cathy Egan, Sarah Ennis, Paul Foster, Marcus Fruttiger, John Gallacher, David (Ted) Garway-Heath, Jane Gibson, Jeremy Guggenheim, Chris Hammond, Alison Hardcastley, Simon Harding, Ruth Hogg, Pirro Hysi, Pearse Keane, Sir Peng Tee Khaw, Anthony Khawaja, Gerassimos Lascaratos, Thomas Littlejohns, Andrew Lotery, Robert Luben, Phil Luthert, Tom Macgillivray, Sarah Mackie, Savita Madhusudhan, Bernadette Mcguinness, Gareth Mckay, Martin Mckibbint, Tony Moore, James Morgan, Eoin O'Sullivan, Richard Oram, Chris Owen, Praveen Patel, Euan Paterson, Tunde Peto, Axel Petzold, Nikolas Pontikos, Jugnoo Rahi, Alicja Rudnicka, Naveed Sattar, Jay Self, Panagiotis Sergouniotis, Sobha Sivaprasad, David Steel, Irene Stratton, Nicholas Strouthidis, Cathie Sudlow, Zihan Sun, Robyn Tapp, Dhanes Thomas, Mervyn Thomas, Emanuele Trucco, Adnan Tufail, Ananth Viswanathan, Veronique Vitart, Mike Weedon, Katie Williams, Cathy Williams, Jayne Woodside, Max Yates, Yalin Zheng

**Affiliations:** School of Optometry & Vision Sciences, Cardiff University, Cardiff, United Kingdom

**Keywords:** Gene-environment interaction, Myopia, Refractive error, vGWAS, vQTL

## Abstract

**Purpose:**

To compare the performance of a novel method for identifying candidate gene-environment interaction loci, Scalable Cauchy Aggregate test using Multiple Phenotypes to test Interactions (SCAMPI) (doi:10.1101/2024.09.10.612314v1), to widely used existing methods, Levene’s test and conditional quantile regression (CQR). The 3 methods were evaluated for refractive error, a trait known to exhibit widespread gene-environment interaction effects.

**Design:**

A cohort study; a genome-wide association study to investigate variance heterogeneity of refractive error.

**Participants:**

A discovery sample of 77 880 UK Biobank participants with records of both refractive error and age of onset of spectacle wear (AOSW) and a validation sample of 257 265 UK Biobank participants with known AOSW.

**Methods:**

SCAMPI, Levene’s tests, and CQR were applied in the discovery sample. Variance quantitative trait loci (vQTLs) identified using SCAMPI were assessed in the independent validation sample. The SCAMPI vQTLs were further assessed for evidence of genotype-by-education interaction or gene–gene interaction, using linear regression.

**Main Outcome Measures:**

Genetic variance heterogeneity associated with refractive error (phenotypic variance differences across genotypes).

**Results:**

SCAMPI identified 15 independent vQTLs with *P* <5.0e-08 while 3 and 11 were found using Levene’s test and CQR, respectively. Of the 15 SCAMPI vQTLs, 12 (80%) were supported in the validation data set after accounting for multiple testing. The lead SCAMPI variants included known vQTL associated with refractive error, such as rs12193556 (*LAMA2*) and rs685352 (*GJD2*), as well as 3 novel candidate gene-environment interaction loci. Among these novel candidates was rs7077247, an intronic variant in the *TCF7L2* gene, which was associated with a –0.085 D greater shift toward myopia in participants with a university degree (*P* = 9.43e-04). This variant is known to be associated with the risk of diabetes and *TCF7L2* is a component of the Wnt signaling pathway. The SCAMPI vQTLs demonstrated minimal evidence for gene–gene interactions.

**Conclusions:**

The SCAMPI outperformed Levene’s test and CQR in identifying candidate gene-environment interaction loci associated with refractive error. Our results suggest that a variant in the *TCF7L2* gene may confer susceptibility to myopia to a greater extent in those with higher vs. lower educational attainment and suggests a molecular link to Wnt signaling and the risk of myopia associated with intensive education.

**Financial Disclosure(s):**

Proprietary or commercial disclosure may be found in the Footnotes and Disclosures at the end of this article.

Myopia is a common eye disease that is anticipated to affect nearly 50% of the population globally by 2050.^1^ Blurred vision in myopia is caused by a mismatch between the refractive power of the eye and its axial length, resulting in the ocular image being positioned in front of the retina. Notably, the excessive elongation of the eye during myopia development is an important risk factor for vision-threatening complications.^2^ For instance, myopia increases the risk of primary open-angle glaucoma and cataract.^3^

The mechanisms underlying myopia onset and progression have not been clearly defined. Epidemiological studies suggest that excessive education/near work activity and insufficient time spent outdoors in childhood are the main environmental risk factors of myopia.^4^^,^^5^ Interventions to increase the time school-aged children spend outdoors reduce the incidence of myopia.^6^ Genetic factors are also implicated in myopia development. For example, monogenic and syndromic forms of myopia have been documented^7^, along with evidence that polygenic inheritance contributes to most cases of ‘common’ myopia.^8^ More than 450 genetic loci independently associated with refractive error have been identified via genome-wide association studies.^9^ However, recent research suggests that the effect of many genetic variants associated with refractive error is modified depending on exposure to specific environmental risk factors such as a person’s level of education, through gene-environment interaction.^10^ For example, an additional copy of the ‘A’ allele of rs121934436 (*LAMA2*) is associated with a –0.13 D greater myopic shift in individuals with a university degree compared with their counterparts without a degree.^11^ The existence of these gene-environment interactions suggests that some individuals are more susceptible to lifestyle risk factors than others, and perhaps that some individuals may benefit more than others from interventions to slow myopia progression.

The most successful strategy for identifying genetic variants with gene-environment interaction effects has been a 2-step approach that first screens variants from across the genome for ‘variance heterogeneity’ with the phenotype-of-interest^12^ (variance heterogeneity refers to a difference in phenotypic variance between individuals carrying 0, 1, or 2 copies of the index variant; such variants are termed, ‘variance quantitative trait loci’ [vQTL]). Then, in the second step, candidate variants from step 1 are directly tested for a gene-environment interaction. The success of the 2-step variance heterogeneity genome-wide association studies (vGWAS) strategy stems from the small number of variants that need to be tested in step 2, compared to the vast number of variants that are tested in step 1 or that need to be tested directly when carrying out a genome-environment wide interaction study using linear regression.^13^ An additional advantage of the 2-step vGWAS approach is that environmental information is not needed for the initial screening step; this allows the method to be applied in large biobanks that have information on phenotypes and genotypes, but little data on lifestyle risk factor exposure. The second step of the vGWAS, with its lower multiple testing burden, can then be applied in smaller patient cohorts that contain information about relevant environmental risk exposure.^14^ The statistical tests most often used to test for variance heterogeneity in step 1 of a vGWAS are Levene’s test and CQR.^12^^,^^15^

Very recently, Bian et al^16^ reported a novel statistical method for genome-wide vQTL screening, called SCAMPI (Scalable Cauchy Aggregate test using Multiple Phenotypes to test Interactions). The authors reasoned that by leveraging information from multiple correlated phenotypes, SCAMPI would be more powerful in detecting vQTL than single-phenotype detection methods such as Levene’s test. SCAMPI fits a regression model relating genotypes to the cross product of pairwise combinations of correlated phenotypes and then aggregates the *P* values from each regression using the Cauchy Combination Test. Bian et al^16^ found that the statistical power of SCAMPI increased as the correlation between phenotypes increased.

The aim of the current study was to evaluate SCAMPI as a method to identify vQTL associated with spherical equivalent refraction (SER). Since Levene’s test and CQR have been used successfully to identify vQTL associated with SER^11^^,^^17^, we performed head-to-head comparisons of SCAMPI with Levene’s test and CQR. The correlated phenotypes used in this study were SER itself and the age of onset of spectacle wear (AOSW); the Pearson correlation coefficient for these 2 traits was *r =* 0.39 (*P* < 2.2e-16) in our discovery data set (described below). Furthermore, we explored the sources of this phenotypic variance heterogeneity associated with refractive error.

## Methods

### Study Cohort and Genotyping

UK Biobank recruited >500 000 participants, aged 40 to 70, from England, Scotland, Wales, and Northern Ireland. Ethical approval for the study was obtained from the National Health Service Research Ethics Committee (Ref 11/NW/0382), and all participants provided written informed consent. The study adhered to the Declaration of Helsinki. At a clinical assessment visit, UK Biobank participants underwent noncycloplegic autorefraction (Tomey RC 5000 autorefractor, Tomey Corp.). Spherical equivalent refraction was calculated by the formula: SER = autorefraction sphere power + 0.5 × autorefraction cylinder power. Spherical equivalent refraction was averaged between the right and left eyes to provide a phenotype value for each individual. If data for both eyes were not available, then the eye with the single record was used instead. Participants self-reported their age of onset of starting to wear spectacles or contact lenses (AOSW) and the age at which they completed full-time education (“EduAge”), except for those with a higher degree. Therefore, individuals with a university or college degree were categorized as completing 21 years of education. Those who reported completing their full-time education before 15 years old were recorded as having completed education at age 15 years, while those who reported completing their education beyond age 21 years were recorded as completing 21 years, to account for low participant counts beyond these values. A binary educational classification (“UniEdu”) was also considered in the analyses; specifically, participants with a university or college degrees were coded as 1, or otherwise coded as 0. The DNA samples from UK Biobank participants were genotyped using 1 of 2 single nucleotide polymorphism (SNP) arrays: either the UK BiLEVE Axiom array or the UK Biobank Axiom array. Variant imputation was completed using a joint Haplotype Reference Consortium + UK 10,000 Genomes Project reference panel.^18^ Approximately 1 million HapMap3 variants were analyzed in the current study.

**Figure 1:**
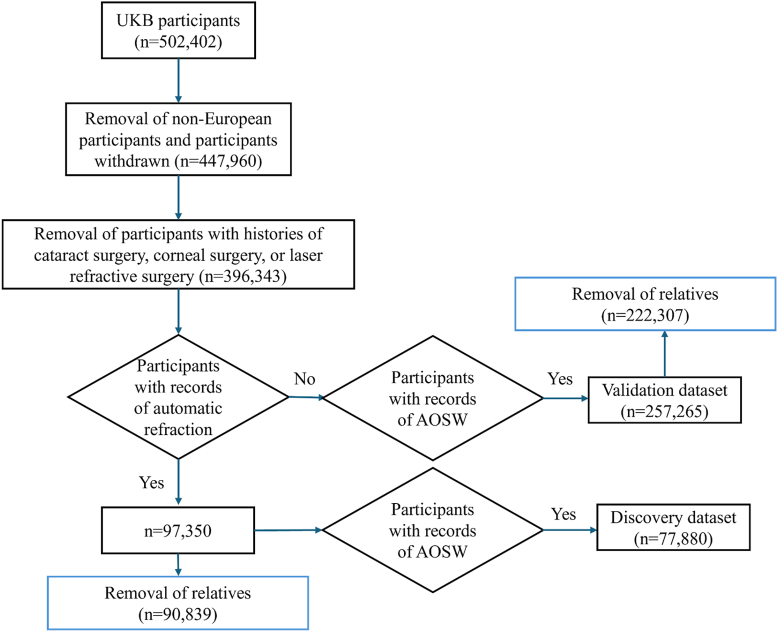
Sample selection for the study. AOSW = age of onset of spectacle wear; UKB = UK Biobank.

After excluding participants who had withdrawn consent to remain in the study, we additionally excluded participants whose genetic heterozygosity was beyond 10 standard deviations from the mean, along with participants whose genetic ancestry based on principal components (PCs) 1 and 2 were beyond 10 standard deviations of the mean of self-reported ‘White British’ participants. Any participant with a medical history of ocular surgery that potentially affected refraction status was also removed. Specifically, we considered a history of cataract surgery (data field: 41200, C711-712, C718-719, C723, C751), corneal surgery (data field: 41200, C442, C444, C445, C448, C461-463, C465, C493), and self-reported refractive laser surgery (data field: 5325). We initially selected participants who had records for both SER and AOSW. This resulted in a sample of 77 880 participants, which was used as a ‘discovery’ data set. Next, the remaining 257 265 participants who had information for AOSW but not SER and who were unrelated to any participant in the discovery data set were selected as a ‘validation’ data set (Fig 1). Samples of unrelated participants were used for exploring interaction effects; only 1 individual was retained in the sample for pairs with a kinship coefficient at least 0.04.

### Variance Heterogeneity Analysis with SCAMPI, Conditional Quantile Regression, and Levene’s Test

Since Levene’s test cannot handle covariates and because CQR analysis is faster in the absence of covariates, the SER and AOSW phenotypes were each regressed on the covariates, sex, age, squared age, genotype array, and PC1-10 prior to the Levene’s test and CQR vGWAS analyses. For the SCAMPI vGWAS analysis, the covariates sex, age, squared age, genotype array, and PC1-10 were included in the analysis rather than being regressed out beforehand. Phenotypes were not transformed to a normal distribution prior to analysis, since preliminary tests suggested this impaired the performance of SCAMPI, CQR, and Levene’s test in detecting *bona fide* vQTLs. Outlier phenotype values were not removed; Levene’s median test and CQR are robust to outliers,^15^^,^^19^ and we were interested to test whether SCAMPI was similarly robust.

The R package SCAMPI (https://github.com/epstein-software/SCAMPI) was run using the default settings. HapMap3 variants (https://doi.org/10.5281/zenodo.7773502) were allocated to chunks of 4000 variants; for the discovery data set of approximately n = 77 880 participants, SCAMPI required 50 h and 10 GB memory on a single computer processing unit to analyze each chunk (R version 4.4.0). By contrast with Levene’s test and CQR, SCAMPI leverages information from >1 phenotype. Pleiotropic gene-environment interaction loci associated with 2 or more correlated phenotypes not only induce a difference in the variance of each trait across genotype categories, they also induce covariance patterns between the traits.^20^ Bian et al^16^ derived an equation to quantify the correlation between 2 phenotypes influenced by a common gene-environment interaction; they then reparametrized the equation so that it could be fit using only trait values and SNP genotypes in a set of individuals. The approach was further extended to handle 2 or more correlated traits, resulting in the SCAMPI analysis framework. SCAMPI outputs a variance heterogeneity *P* value aligned to each input trait, as well as an aggregate *P* value from the Cauchy combination test that indicates whether a variant was associated with at least one of the covariance patterns between pairs of input traits. In the current work, we restricted attention to the “P1” variance heterogeneity *P* value aligned with the SER phenotype (for comparison, we describe an exploratory analysis of the aggregate *P* values obtained using the SCAMPI Cauchy combination test in Supplementary Note 1).

Conditional quantile regression was performed for the SER phenotype in the discovery data set at 9 quantile values (0.1, 0.2, 0.3, … 0.9), using the rq function from the R package quantreg version 6.1. HapMap3 SNPs were processed in chunks of 4000 SNPs using the snp_readBed2 function from the R package bigsnpr version 1.12.18. Conditional quantile regression analysis took approximately 16 h and 1 GB memory on a single computer processing unit to analyze each chunk of 4000 variants. Following Pozarickij et al,^17^ the SNP regression coefficients ($\beta_{SNP}$) at the 9 quantiles (τ) were fit with an inverse variance-weighted restricted maximum likelihood meta-regression model that included an intercept term, a linear term ($\beta_{SNP}\times \tau$) and a quadratic term ($\beta_{SNP}\times \tau^{2}$) using the rma function from the R package metafor version 4.8-0. The variance heterogeneity *P* values from the quadratic term were taken forward for further analysis.^17^ Levene’s median test was implemented in the OmicS-data-based Complex trait Analysis genetic analysis software program, using the --vqtl option.^21^ By contrast with SCAMPI, the only input trait for the CQR analysis and the Levene’s test analysis was the SER phenotype residuals.

### Genomic Control and Clumping

A genomic inflation factor (λ_GC_) was calculated for the SCAMPI, Levene’s test, and CQR analyses of the discovery data set. A λ_GC_ > 1 is indicative of an excess of low *P* values caused by factors such as relatedness or a non-normal phenotype distribution.^22^ To adjust *P* values, the χ^2^ statistic was calculated for each variant according to its original *P* value; an adjusted *P* value was calculated^22^ after dividing the χ^2^ by λ_GC_. PLINK (v1.9)^23^ was used to identify independent genome-wide significant variants, using the parameters: *--clump-p* 5e-08; *--clump-kb* 1000; *--clump-r2* 0.1.

### Validation of Variants Found in the Discovery Data Set

The 15 independent vQTL identified by SCAMPI were assessed in the validation data set. Since participants in the validation data set only had information for AOSW, Levene’s test was used to assess the variance heterogeneity of residual AOSW after adjusting for the covariates used in the discovery analysis. A Bonferroni *P* value of *P* < 0.003 (0.05/15) was regarded as a successful validation.

### Exploring Sources of Phenotypic Variance Heterogeneity

Linear regression was used to test for gene-environment or gene–gene interactions associated with SER and AOSW. First, we tested each of the 15 lead SCAMPI variants for an interaction associated with SER in a sample of unrelated participants in UK Biobank with an SER record (Fig 1, blue frame; n = 90 839). Then we tested the 15 variants for an interaction associated with AOSW in a sample of unrelated participants with an AOSW record who were not included in the SER analysis (Fig 1, blue frame; n = 222 307). For the gene-environment interaction tests, we performed linear regression analyses testing for an interaction between SNP genotype and either UniEdu or EduAge (Equations (1a), (1b), (2a), (2b)). Here, β_0_ is an intercept term; β_1_ and β_2_ are coefficients representing the main effects of the variant and UniEdu, respectively; β_3_ is a genotype-by-education interaction term; γ represents the coefficients of covariates (age, squared age, sex, genotype array, and PC1-10); and ε are normally distributed residuals. A Bonferroni-adjusted *P* value for the β_3_ term of *P* < 0.003 (0.05/15) was regarded as statistically significant. Gene–gene interactions were tested using Equations (3), (4). Here, β_1_ and β_2_ represent the main effect of each variant, while the β_3_ coefficient is a genotype-by-genotype interaction term. A Bonferroni-adjusted *P* value for the β_3_ term of *P* < 4.8e-04 (0.05/105 pair-wise interactions) was regarded as statistically significant. Gene-environment correlations were examined using Equations (5a), (5b). If *P* < 0.05 for the β_1_ term representing gene-environment correlation, then any gene-environment interaction involving that variant was considered a potential false-positive finding.

(1a) $$SER=\beta_{0}+\beta_{1}G+\beta_{2}UniEdu+\beta_{3}G\times UniEdu+\gamma C+\varepsilon$$

(1b) $$SER=\beta_{0}+\beta_{1}G+\beta_{2}EduAge+\beta_{3}G\times EduAge+\gamma C+\varepsilon$$

(2a) $$AOSW=\beta_{0}+\beta_{1}G+\beta_{2}UniEdu+\beta_{3}G\times UniEdu+\gamma C+\varepsilon$$

(2b) $$AOSW=\beta_{0}+\beta_{1}G+\beta_{2}EduAge+\beta_{3}G\times EduAge+\gamma C+\varepsilon$$

(3) $$SER=\beta_{0}+\beta_{1}G_{1}+\beta_{2}G_{2}+\beta_{3}G_{1}\times G_{2}+\gamma C+\varepsilon$$

(4) $$AOSW=\beta_{0}+\beta_{1}G_{1}+\beta_{2}G_{2}+\beta_{3}G_{1}\times G_{2}+\gamma C+\varepsilon$$

(5a) $$UniEdu=\beta_{0}+\beta_{1}G+\gamma C+\varepsilon$$

(5b) $$EduAge=\beta_{0}+\beta_{1}G+\gamma C+\varepsilon$$

In addition to investigating interactions between educational parameters and SNP genotypes, we also investigated interactions relating to the demographic characteristics sex and age. For these analyses, linear regressions were modeled using the participants’ age or sex in place of UniEdu in Equations (1a), (2a).

Nonadditive genetic effects, for example, a dominant or recessive effect, could be another reason for the observed variance heterogeneity. We used a previously described method^24^ to investigate whether any of the 15 lead variants had a nonadditive association with SER or AOSW. Here, a ‘dominance deviation’ term was added alongside the additive genetic term in linear regression models, in which the heterozygous genotype was coded as “1,” and otherwise coded as “0” (Equations (6a), (6b)). A *P* value for the dominance deviation term ($\beta_{2}$) of *P* < 0.003 (0.05/15) was taken as significant evidence of a nonadditive effect.

(6a) $$SER=\beta_{0}+\beta_{1}G+\beta_{2}G_{dominant}+\gamma C+\varepsilon$$

(6b) $$AOSW=\beta_{0}+\beta_{1}G+\beta_{2}G_{dominant}+\gamma C+\varepsilon$$

## Results

### Demographic Characteristics of Participants

The demographic characteristics of the discovery and validation populations were highly comparable (Table 1). The mean age in both data sets was approximately 58 years, and about 54% of participants were female. The median AOSW was the same in both data sets (39 years). The mean SER in the discovery data set was –0.30 D (standard deviation = 2.78). The proportion of the sample having a university or college degree was higher in the discovery data set than the validation data set (38.0% vs. 33.6%, respectively; chi-squared test, *P* < 2.20e-16).

**Table 1:** Demographic Characteristics of Participants in the Discovery and Replication Data Sets

Demographic Characteristic	Discovery Data Set (n = 77 880)	Replication Data Set (n = 257 265)
Age (years)	58.79 (7.47)	57.89 (7.57)
Sex (percentage female)	53.48%	54.73%
SER (D)	–0.30 (2.78)	NA
AOSW (years)	39 (15, 47)	39 (15, 47)
EduAge (years)	18.30 (2.51)	18.00 (2.53)
UniEdu (percentage)	37.96%	33.61%

AOSW = age of onset of spectacle wear; D = diopters; EduAge = the age of completed full-time education; NA = not applicable; SD = standard deviation; SER = spherical equivalent refraction; UniEdu = university or college degree.

Values presented as mean (SD), except for AOSW, which is shown as median (Q1, Q3).

### Comparison of SCAMPI, Levene’s Test, and CQR Analyses

**Figure 2:**
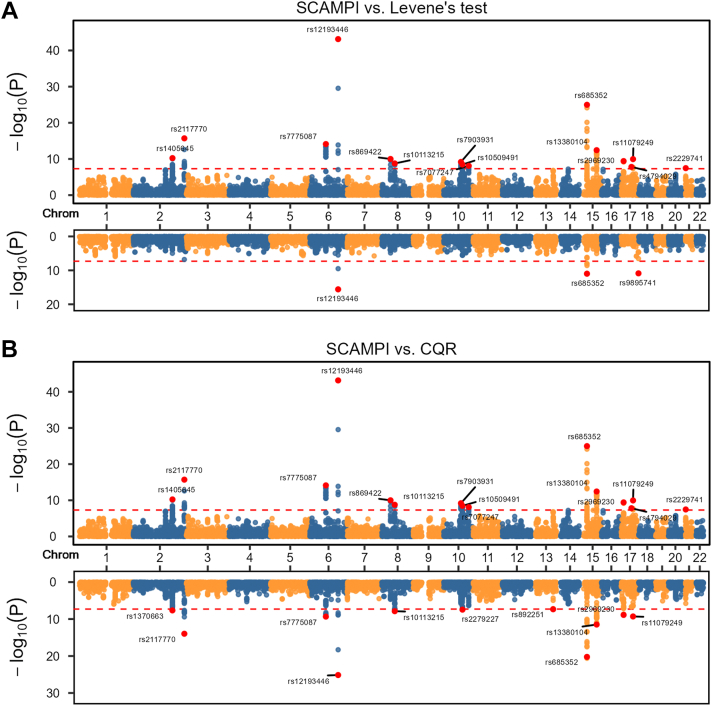
Miami plots comparing SCAMPI and Levene’s test or CQR. (**A**) Upper panel shows results for the SCAMPI vGWAS. The 15 independent vQTLs identified by SCAMPI are highlighted in red. Lower panel shows results for the Levene’s test vGWAS. The 3 independent vQTLs identified by Levene’s test are highlighted in red. (**B**) Upper panel shows results for SCAMPI vGWAS, as in panel A. Lower panel shows results for the CQR vGWAS, which identified 11 independent vQTLs, as highlighted in red. For all plots, the x-axis represents genomic position on the specified chromosome; y-axes represent statistical significance in the vGWAS analysis. The horizontal red dashed line in each plot indicates the genome-wide significance level (*P* = 5e-08, corresponding to -log_10_(*P*) = 7.3). CQR = conditional quantile regression; vGWAS = variance heterogeneity genome-wide association study; vQTL = variance heterogeneity quantitative trait locus.

SCAMPI demonstrated a genomic inflation factor of λ_GC_ = 1.18, which suggested a moderate, systematic excess of low *P* values, potentially arising from the inclusion of relatives in the discovery data set, the non-normal distributions of SER and AOSW, or true polygenicity. Prior studies have used genomic control^22^ to adjust for the inflation of test statistics in vGWAS analyses.^25^^,^^26^ Therefore, we report results after genomic control correction. Of the approximately 1 million HapMap3 variants examined using SCAMPI, 138 displayed genome-wide significant evidence of variance heterogeneity associated with SER, prior to clumping. Fifteen independently associated vQTLs remained after clumping (Fig 2A, upper panel). Levene’s test exhibited a slightly lower level of genomic inflation compared to SCAMPI: λ_GC_ = 1.10. After applying genomic control to adjust for this inflation, Levene’s test identified 8 variants associated with SER variance heterogeneity prior to clumping and 3 independent vQTLs after clumping (Fig 2A, bottom panel). Two vQTLs were genome-wide significant using both methods: an intronic variant of *LAMA2* (rs12193446) and a variant upstream of *GJD2* (rs685352). SCAMPI provided stronger evidence of association for both variants (rs12193446: *P*_*SCAMPI*_ = 7.13e-44 vs. *P*_*Levene*_ = 2.56e-16; rs685352: *P_SCAMPI_* = 1.06e-25 vs. *P_Levene_* = 1.02e-11). The only vQTL identified by Levene’s test (*P* = 1.32e-11) but not by SCAMPI (*P* = 0.41) was rs9895741 located in the *NPLOC4*/*TSPAN10* region.

**Figure 3:**
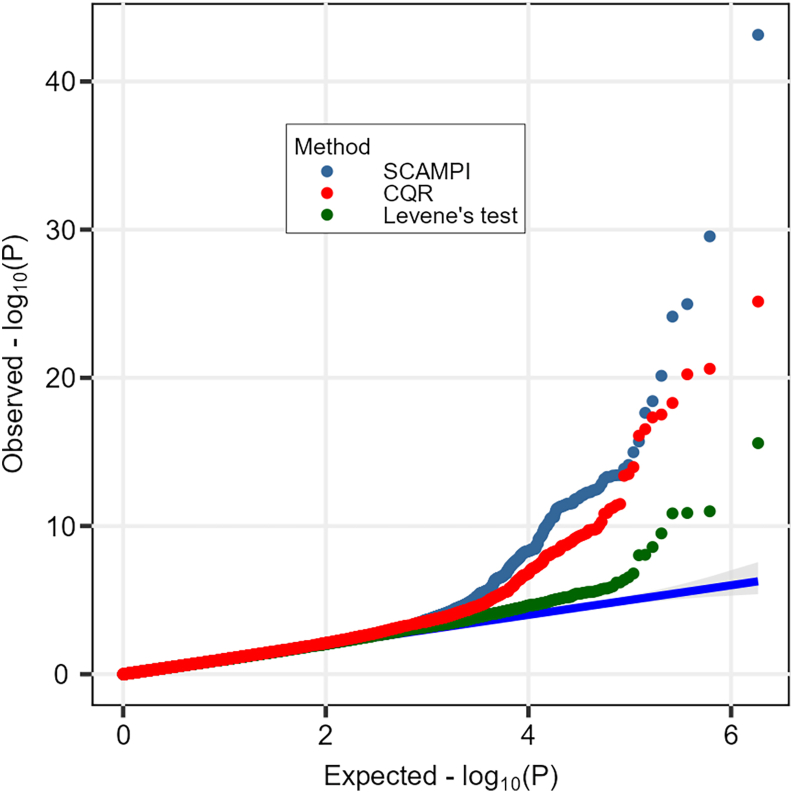
Q-Q plot for the 3 vGWAS methods. The solid blue line represents the observed vs. expected –log_10_(*P*) values under the null hypothesis, while the gray shaded region is the 95% confidence interval for the null hypothesis. The blue, red, and green data points are the vGWAS *P* values in the discovery data set for SCAMPI, CQR, and Levene’s test, respectively, after genomic control correction. CQR = conditional quantile regression; vGWAS = variance heterogeneity genome-wide association study.

**Figure 4:**
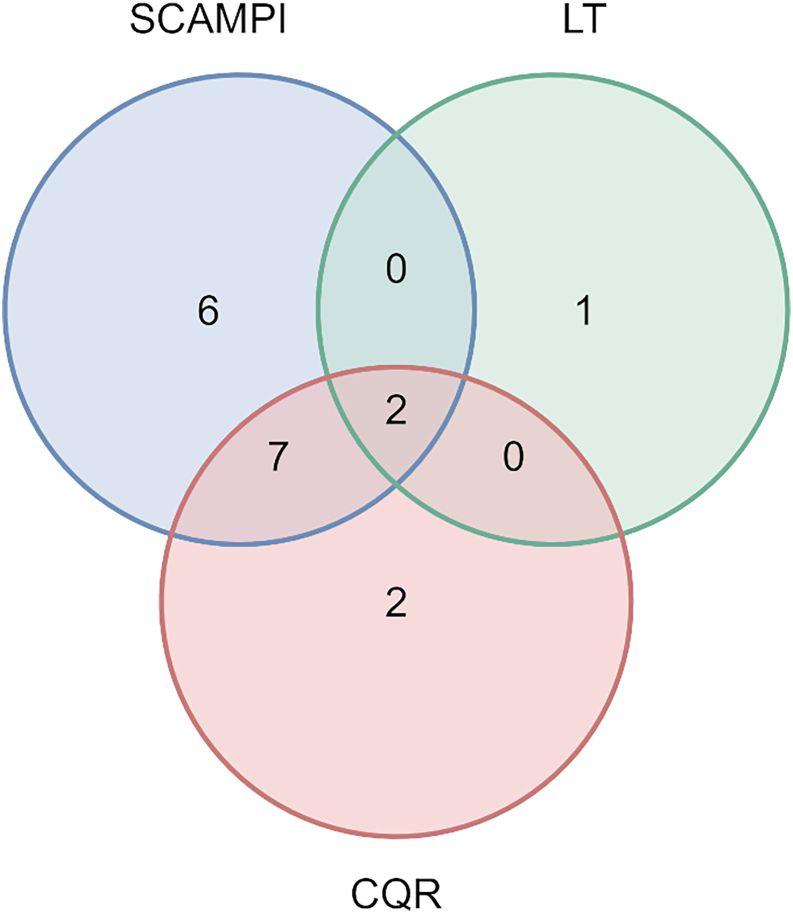
Venn diagram illustrating the number of independent vQTL identified by SCAMPI, Levene’s test, and CQR. CQR = conditional quantile regression; LT = Levene’s test.

Conditional quantile regression also revealed slight inflation, λ_GC_ = 1.13, however less than SCAMPI. Conditional quantile regression detected 76 genome-wide significant variants prior to clumping, of which 11 were left after clumping. Most (9/11) of the variants identified by CQR were also identified by SCAMPI (Fig 2B), in which, rs683922 was in linkage disequilibrium (LD) with rs685352 (*r*^2^ = 0.94), rs1370663 was in LD with rs1405645 (*r*^2^ = 0.23). Two variants were uniquely identified by CQR: rs2279227 (RGR) and rs892251 (LINC0045). These 2 variants failed to reach genome-wide significance in either the SCAMPI or Levene’s test analysis (rs2279227: *P*_*SCAMPI*_ = 2.18e-07 vs. *P*_*Levene*_ = 3.70e-03; rs892251: *P_SCAMPI_* = 1.60e-07 vs. *P_Levene_* = 9.57e-03). A quantile–quantile (Q-Q) plot supported the conclusion that SCAMPI was more powerful than Levene’s test and CQR in finding vQTLs associated with refractive error (Fig 3, Supplementary Figure). Two variants (rs12193446 and rs685352) were identified by all 3 methods (Fig 4).

Twelve of the 15 vQTLs identified by SCAMPI were successfully validated as vQTLs in the validation data set (*P* < 0.003; Bonferroni correction for testing 15 variants, Supplementary Table S1, available at www.ophthalmologyscience.org) providing a validation success rate of 80%. One additional variant, rs4794029 (*GNGT2*), reached nominal significance. Detailed results for the 15 SCAMPI variants can be found in Table 2. All of the genes annotated to these variants were previously reported to be associated with SER, consistent with the hypothesis that gene-environment interactions play an important role in myopia development.^17^ Notably, 9 of the SCAMPI vQTL (*RASGRF1*, *PDE11A*, *NOG*, *SHISA6*, *KCNMA1*, *CDHR1*, *TCF7L2*, *GNGT2*, and *ASMER1*) were not detected in a recent study by Clark et al^11^ that used Levene’s test to screen for refractive error vQTLs. However, several of the SCAMPI vQTLs were known gene-environment interaction variants or were in high LD with such variants. For instance, rs7775087 (*KCNQ5*) is in high LD (*r*^*2*^ = 0.82) with rs7744813, which was shown to interact with educational attainment in its association with SER.^11^ Similarly, rs869422 (*ZMAT4*) is in high LD (*r*^*2*^ = 0.97) with rs7829127, which was also reported to interact with education level and near work to influence myopia.^27^

**Table 2:** Discovery, Replication, and Comparison of Significant vQTLs for SER Found by SCAMPI

rsID	CHR	BP	A1	A2	AF	*P*_SCAMPI_	*P*_*CQR*_	*P*_Levene_	*P*_replication_	Annotation
rs12193446	6	129820038	G	A	0.096	7.13E-44	7.12E-26	2.56E-16	1.40E-29	*LAMA*
rs685352	15	35008335	G	A	0.45	1.60E-25	5.79E-21	1.02E-11	7.20E-09	*GJD2*
rs2117770	2	233375784	T	C	0.29	1.98E-16	1.07E-14	1.60E-07	2.36E-08	*PRSS56*
rs7775087	6	73606783	G	T	0.44	7.58E-15	4.62E-10	2.55E-04	2.68E-09	*KCNQ5*
rs13380104	15	79378821	T	C	0.42	3.73E-13	3.29E-12	6.95E-05	6.98E-05	*RASGRF1*
rs1405645	2	178853378	G	A	0.46	5.78E-11	2.80E-17	2.71E-03	4.78E-10	*PDE11A*
rs869422	8	40723970	G	A	0.21	1.01E-10	2.12E-05	6.58E-05	7.65E-13	*ZMAT4*
rs11079249	17	54716686	A	G	0.36	1.10E-10	5.02E-10	2.12E-02	4.02E-01	*NOG*
rs2969230	17	11419528	C	T	0.48	3.88E-10	1.32E-09	6.24E-03	6.45E-05	*SHISA6*
rs7903931	10	79114690	C	T	0.36	6.64E-10	3.63E-06	3.71E-03	7.56E-04	*KCNMA1*
rs10113215	8	60132194	G	A	0.33	1.76E-09	1.35E-08	1.02E-04	1.00E-12	*TOX*
rs10509491	10	85977175	A	G	0.47	3.44E-09	6.93E-08	2.86E-06	2.27E-06	*CDHR1*
rs7077247	10	114812071	C	T	0.46	7.93E-09	8.37E-07	2.20E-03	2.42E-01	*TCF7L2*
rs4794029	17	47280301	T	C	0.32	1.56E-08	2.16E-07	1.40E-01	2.88E-02	*GNGT2*
rs2229741	21	16340289	T	C	0.42	3.45E-08	3.00E-05	1.20E-01	1.31E-04	*ASMER1*

A1 = effect allele; A2 = noneffect allele; AF = allelic frequency of effect allele; BP = physical position of variant (refer to hg19 assembly); CHR = chromosome; CQR = conditional quantile regression.

Variants located outside genes (rs685352, rs2117770, rs11079249, and rs10113215) were annotated to their nearest genes. Ordering according to the significance of each vQTL.

### Gene-Environment and Gene–Gene Interactions of the 15 Lead SCAMPI Variants

**Figure 5:**
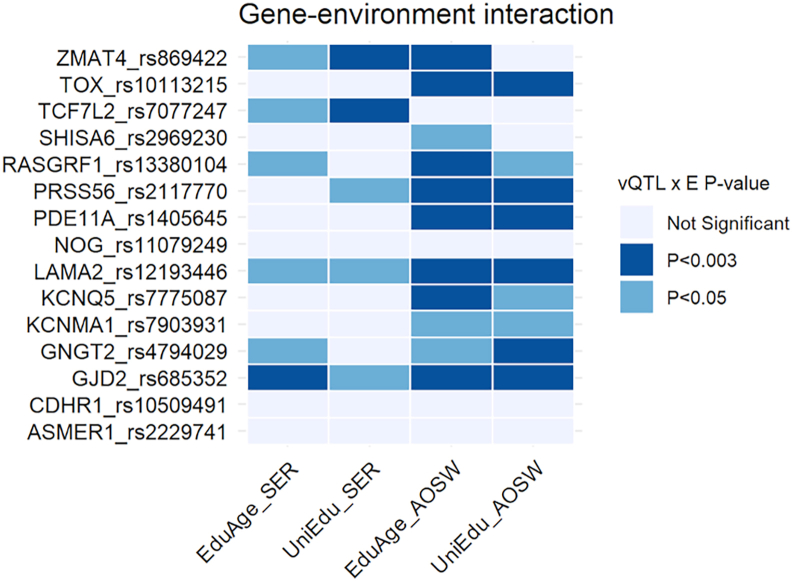
Summary of genotype-by-education interactions associated with SER and AOSW. The color of each cell represents the statistical significance (*P* value) of a test for gene-environment interaction. The first 2 columns show interactions associated with SER. The last 2 columns show interactions associated with AOSW. AOSW = age of onset of spectacle wear; E = environments; EduAge = age completed full-time education; SER = spherical equivalent refraction; UniEdu = binary educational levels; vQTL = variance heterogeneity quantitative trait locus.

The 15 SCAMPI variants were directly tested for an interaction with UniEdu using linear regression analysis, with either SER or AOSW as the outcome phenotype (Table 3; Fig 5). In the SER analysis, 5 vQTLs reached at least nominal statistical significance; more than expected by chance (*P* = 6.14e-04). For the larger AOSW analysis, 11 vQTLs reached at least nominal statistical significance, again more than expected by chance (*P* = 5.53e-12). After accounting for testing 15 variants, 2 and 6 of the SCAMPI vQTLs displayed significant evidence of a genotype-by-UniEdu interaction with the SER and AOSW phenotypes, respectively. As expected given the very high correlation of UniEdu and EduAge, similar results were obtained when testing for a genotype-by-EduAge interaction (Supplementary Table S2, available at www.ophthalmologyscience.org; Fig 5). Three loci displayed evidence of an interaction with educational attainment for the first time, to the best of our knowledge. For the SER phenotype, the ‘C’ allele of rs7077247—an intronic variant in *TCF7L2*—was associated with a –0.085 D (*P* = 9.43e-04) greater shift toward myopia in individuals with a university or college degree. For the AOSW phenotype, the ‘G’ allele of rs1405645 (P*DE11A*), and the ‘C’ allele of rs4794029 (*GNGT2*) were associated with a 0.35 year (*P* = 1.05e-03), and 0.40 year (*P* = 5.51e-04) earlier age of starting to wear spectacles in those with a university or college degree.

**Table 3:** Gene-UniEdu Interactions Associated with SER and AOSW for the 15 SCAMPI Lead Variants

rsID	CHR	BP	A1	A2	AF	Annotation	Phenotype: SER			Phenotype: AOSW		
							BETA	SE	P	BETA	SE	P
rs12193446	6	129820038	G	A	0.096	*LAMA*	0.099	0.043	**2.31e-02**	0.76	0.18	**2.23e-05**
rs685352	15	35008335	G	A	0.45	*GJD2*	–0.057	0.026	**2.62e-02**	–0.48	0.11	**8.44e-06**
rs2117770	2	233375784	T	C	0.29	*PRSS56*	–0.063	0.028	**2.67e-02**	–0.40	0.12	**6.95e-04**
rs7775087	6	73606783	G	T	0.44	*KCNQ5*	2.84e-02	0.026	2.73e-01	0.28	0.11	**9.58e-03**
rs13380104	15	79378821	T	C	0.42	*RASGRF1*	–0.043	0.026	9.95e-02	–0.28	0.11	**8.30e-03**
rs1405645	2	178853378	G	A	0.46	*PDE11A*	–0.049	0.026	5.79e-02	–0.35	0.11	**1.05e-03**
rs869422	8	40723970	G	A	0.21	*ZMAT4*	0.11	0.032	**7.48e-04**	0.23	0.13	8.50e-02
rs11079249	17	54716686	A	G	0.36	*NOG*	8.26e-03	0.027	7.58e-01	0.061	0.11	5.82e-01
rs2969230	17	11419528	C	T	0.48	*SHISA6*	0.027	0.026	2.96e-01	–0.19	0.11	8.03e-02
rs7903931	10	79114690	C	T	0.36	*KCNMA1*	–0.015	0.027	5.69e-01	–0.32	0.11	**4.08e-03**
rs10113215	8	60132194	G	A	0.33	*TOX*	0.027	0.027	3.12e-01	0.55	0.11	**1.28e-06**
rs10509491	10	85977175	A	G	0.47	*CDHR1*	0.045	0.026	7.98e-02	0.059	0.11	5.81e-01
rs7077247	10	114812071	C	T	0.46	*TCF7L2*	–0.085	0.026	**9.43e-04**	–0.12	0.11	2.81e-01
rs4794029	17	47280301	T	C	0.32	*GNGT2*	0.043	0.028	1.21e-01	0.40	0.11	**5.51e-04**
rs2229741	21	16340289	T	C	0.42	*ASMER1*	1.93e-03	0.026	9.41e-01	–0.10	0.11	3.36e-01

A1 = effect allele; A2 = noneffect allele; AF = allelic frequency of effect allele; AOSW = age of onset of spectacle wear; BETA = effect size for variant-by-education interaction; BP = physical position of variant (genome build GRCh37; hg19); CHR = chromosome; SE = standard error; SER = spherical equivalent refraction; UniEdu = binary educational levels; vQTL = variance quantitative locus.

Variants located outside genes (rs685352, rs2117770, rs11079249, and rs10113215) were annotated to their nearest genes. Bold *P* values highlighted vQTLs, which were at least nominally significant (*P* < 0.05). Ordering according to the significance of each vQTL in SCAMPI analysis.

In contrast to the high proportion of vQTLs suggestive of involvement in gene-environment interactions, there was little evidence of gene–gene interactions in pairwise tests of the 15 vQTLs. Only variants rs2117770 (*PRSS56*) and rs685352 (GJD*2*) showed strong evidence of an interaction associated with AOSW, which withstood correction for multiple testing (β_3_ = –0.33, *P* = 3.00e-05). Yet there was little statistical support for the interaction when this pair of vQTLs was tested for an interaction in association with SER (*P* = 0.54). Only one variant demonstrated nominal evidence of gene-education correlation in association with SER: rs2229741 (AS*MER1*; *P* = 0.012).

### Genotype-by-Sex and Genotype-by-Age Interactions of the 15 SCAMPI Variants

As shown in Supplementary Table S3, available at www.ophthalmologyscience.org, we found suggestive evidence that rs10509491 (*CDHR1*) differed in its association with SER in male vs. female participants (interaction *P* = 5.53e-04). The *CDHR1* variant was associated with SER in both sexes, but the effect was larger in females (males: β = –0.056, *P* = 1.44e-03; females: β = –0.14, *P* = 3.97e-16). However, this difference in effect size between male and female participants was not replicated in the larger AOSW analysis (*P* = 0.70), suggesting it may be a false-positive finding. Considering potential interactions with age (Supplementary Table S4, available at www.ophthalmologyscience.org), the strongest support was for rs7775087 (*KCNQ5*) in the AOSW analysis (genotype-by-age interaction, β = –0.019 years, *P* = 4.94e-03); however, the association was no longer significant after accounting for multiple testing.

### Nonadditive Genetic Effects of 15 SCAMPI Variants

Six variants showed at least nominal evidence of nonadditive genetic effects associated with SER, more than expected by chance (Supplementary Table S5, available at www.ophthalmologyscience.org; *P* = 5.28e-05). Two of these nonadditive vQTL associations confirmed earlier reports^24^; namely, *ZMAT4* (*P* = 7.99e-07) and *LAMA2* (*P* = 0.019). The other 4 variants with nominal evidence of nonadditive effects were rs685352 (*GJD2*, *P* = 6.37e-03), rs2117770 (*PRSS56*, *P* = 2.09e-02), rs7775087 (*KCNQ5*, *P* = 4.08e-02), and rs2969230 (*SHISA6*, *P* = 1.93e-02). For the AOSW phenotype, only rs869422 (*ZMAT4*) had suggestive significance of a nonadditive association (*P* = 4.89e-03).

## Discussion

Our study suggested that the new method SCAMPI was more powerful than Levene’s test and CQR in identifying vQTLs associated with refractive error. Although SCAMPI demonstrated slightly greater inflation of test statistics than Levene’s test and CQR (λ_GC_ = 1.18 vs. λ_GC_ = 1.10 and 1.13, respectively), after accounting for this via genomic control, SCAMPI detected fivefold more vQTLs compared with Levene’s test (15 vs. 3 vQTLs) and 4 more variants than CQR (15 vs. 11 vQTLs). Furthermore, most vQTLs (2/3) found by Levene’s test and CQR (9/11) were also identified by SCAMPI. Follow-up analyses revealed several vQTLs that could be traced to involvement in gene-environment interactions related to educational attainment, consistent with earlier studies.^11^^,^^27^ Support for other causes of variance heterogeneity such as gene-gene, gene-age, and gene-sex interaction was less compelling.

In analyses of the SER phenotype, variants within or near *ZMAT4*, *TCF7L2*, *PRSS56*, *GJD2*, and *LAMA2* all showed at least nominal evidence of a genotype-by-UniEdu interaction. Variant rs7077247 in *TCF7L2* was particularly noteworthy; although *TCF7L2* has been associated with refractive error in genome-wide association studies analyses,^9^ this is the first time it has been identified as having an interaction effect (genotype-by-UniEdu interactions have been reported previously for *ZMAT4*, *PRSS56*, *GJD2*, and *LAMA2*^11^^,^^27^). *TCF7L2* encodes a transcription factor that binds to DNA through its high-mobility group domain to play a key role in the Wnt signaling pathway. Variants in *TCF7L2* such as rs7077247 are associated with an increased risk of type 2 diabetes across a range of ethnicities.^28^ The SCAMPI vQTL rs7077247 is in moderate LD (*r*^*2*^ = 0.43) with *TCF7L2* variant rs7903146, which has been associated with the risk of developing diabetic retinopathy and retinal microvascular changes.^29^^,^^30^ Animal studies suggest the expression of *TCF7L2* is upregulated in retinal ganglion cells and the inner nuclear layer of a mouse model of diabetic retinopathy, which promotes endoplasmic reticulum stress and enhances endothelium permeability.^31^ Nevertheless, while the *ZMAT4*, *PRSS56*, *GJD2*, and *LAMA2* vQTL also demonstrated confirmatory evidence for a genotype-by-UniEdu interaction in association with AOSW, this interaction did not reach the nominal significance level for the *TCF7L2* variant (*P* = 0.12). Therefore, definitive evidence that education modifies the risk of myopia conferred by the *TCF7L2* vQTL will require further validation in independent cohorts.

What biological mechanism may underlie the link between *TCF7L2* expression, education and myopia? *TCF7L2* is a downstream target of HIF-1α, and upregulation of *TCF7L2* gene and protein expression has been observed in several cell lines under hypoxic conditions.^32^, ^33^, ^34^ Scleral hypoxia and activation of HIF-1α are implicated in myopia development.^35^^,^^36^ Hence, children carrying the rs7077247 risk allele may show an amplified level of hypoxia-induced scleral *TCF7L2* expression during myopia development compared to children who do not carry the risk allele. Thus, the rs7077247 risk allele could amplify the scleral hypoxia response in children developing myopia through an association with intensive education.^37^ However, a more complex molecular interplay between *TCF7L2* and other genes in the canonical Wnt pathway may exist. For example, signaling via the Wnt7b pathway may upregulate MMP-2 activity to promote scleral remodeling.^38^

In addition to testing for an interaction with education, we also explored other potential causes of phenotypic variance heterogeneity. For example, we found suggestive evidence that the ‘G’ allele of rs10509491 (*CDHR1*) interacts with sex to confer a greater risk of myopia in females compared to males. *CHDR1* encodes a photoreceptor-specific cadherin; mutations in this gene have been linked to inherited retinal dystrophy,^39^^,^^40^ and variant rs3814212 in *CHDR1* has previously been associated with refractive error.^40^ There are precedents for genotype-by-sex interactions relating to myopia; for instance, Chen et al^41^ have reported several steroidogenesis enzyme genes that interact with sex to modify the risk of high myopia.

Nonadditive genetic effects, including dominance and recessive genetic effects, are another possible origin of phenotypic variance heterogeneity. Confirming earlier work,^11^ rs869422 (*ZMAT4*) demonstrated strong evidence for a nonadditive association with refractive error (as well as an interaction effect with education). Variance quantitative trait loci within or nearby *GJD2*, *LAMA2*, and *KCNQ5* also had suggestive evidence of nonadditive effects. By contrast, we found little evidence to support epistatic genetic interactions associated with refractive error. One pair of variants (rs2117770 and rs685352) displayed strong evidence of an association with AOSW, but not with SER.

The current work had limitations that should be considered when interpreting the results. First, although AOSW has been used as a proxy for SER in previous studies,^9^^,^^42^^,^^43^ testing for variance heterogeneity of the AOSW phenotype in the validation data set does not constitute direct replication of variance heterogeneity of SER. Second, disparities in the exposure of populations around the world to environmental risk factors for myopia may lead to gene-environment interactions having different effect sizes across study cohorts. Indeed, certain gene-environment interactions associated with refractive error could be unique to a specific study sample. Third, the *TCF7L2* vQTL (rs7077247) was not associated with variance heterogeneity of the AOSW phenotype in the validation data set and, although genotype-by-education interactions at this locus were associated with SER, they were not significantly associated with AOSW. Ideally, the genotype-by-education interaction for the *TCF7L2* vQTL should be replicated in an independent cohort for the SER phenotype. However, apart from UK Biobank, no publicly available large data sets with information for SER and genotype data are available worldwide, to our knowledge.

In summary, SCAMPI performed better than Levene’s test and CQR in detecting vQTLs associated with refractive error, by leveraging the correlation between SER and AOSW. Eighty percent of the SCAMPI vQTL were successfully validated in an independent sample and SCAMPI prioritized several variants and genes already known to be involved in gene-environment interactions related to myopia. The SCAMPI detected a variant in *TCF7L2* that provided novel evidence for a genotype-by-education interaction at this locus. Like other 2-step vQTL approaches for identifying candidate genetic variants involved in gene-environment or gene–gene interactions, SCAMPI benefits from not requiring participant-level information about exposure to lifestyle risk factors. Future work using SCAMPI to search for refractive error vQTL would be enhanced by the inclusion of a wider range of endophenotypes for refractive error, especially axial length, which is highly correlated with refractive error.^44^

## Supplementary Data

Supplementary Note 1
