# Supplementary material for: Gene-Environment Interaction Loci Associated with Refractive Error: SCAMPI Analysis
Source: Ophthalmol Sci. 2026 May 5;6(7):101219. doi: 10.1016/j.xops.2026.101219 (PMC13255065; doi:10.1016/j.xops.2026.101219)
Supplement: Supplementary Note 1 [file mmc1.pdf]

Supplementary Note 1. Results for SCAMPI summary p-values

When analyzing two input phenotypes, SCAMPI outputs a total of four p-values for each genetic marker that is tested. The first three p-values (P1, P2 and P3) relate to regression of cross products on the marker genotype. The fourth p-value is a summary p-value obtained by applying a Cauchy combination test (CCT) to the first three p-values. In our primary analysis, we utilized the P1 p-value from SCAMPI, since although this is based on variance heterogeneity in both input phenotypes, we assumed it was most closely aligned to input trait #1 (SER). However, to provide a more thorough evaluation of SCAMPI, we also performed an exploratory analysis of the summary (CCT-based) p-values.

After genomic control correction and clumping, the SCAMPI CCT p-values obtained in the discovery dataset identified a total of 17 independent vQTLs ($P < 5.0e\text{-}08$). Fifteen of the 17 SCAMPI CCT vQTLs were the same as the 15 independent vQTLs identified using the SCAMPI P1 p-values (Table 2). Details of the two novel vQTLs (rs2358481 and rs7163368) are shown in the table below.

rsID	CHR	POS	REF	ALT	MAF	*P*¶	Nearest gene(s)
rs2358481	14	54672272	C	T	0.318	4.46e-08	LOC105370505 / CDKN3
rs7163368	15	63571121	T	C	0.217	4.12e-08	APH1B

¶ SCAMPI CCT p-value

Both of the novel vQTLs identified using the SCAMPI CCT p-values were validated as vQTL in the validation dataset.

rsID	CHR	POS	*Validation dataset P*
rs2358481	14	54672272	3.93e-04
rs7163368	15	63571121	3.21e-04
