# Supplementary material for: Gene-Environment Interaction Loci Associated with Refractive Error: SCAMPI Analysis
Source: Ophthalmol Sci. 2026 May 5;6(7):101219. doi: 10.1016/j.xops.2026.101219 (PMC13255065; doi:10.1016/j.xops.2026.101219)
Supplement: Supplementary Table 1 [file mmc2.pdf]

Supplementary Table 1. Discovery and replication of vQTLs for AOSW found by SCAMPI

rsID	CHR	BP	A1	A2	AF	P_{SCAMPI}	$P_{replication}$	Annotation
rs6000342	22	36989950	G	A	0.008	1.38E-22	2.08E-01	*CACNG2*
rs11985370	8	123909890	A	G	0.008	7.37E-17	6.33E-01	*ZHX2*
rs1917357	7	124268066	T	C	0.010	6.51E-12	2.28E-01	*POT1*
rs9895741	17	79603831	A	G	0.36	3.76E-09	6.21E-67	*NPLOC4*
rs13375303	1	238400917	C	A	0.010	5.28E-09	3.93E-01	*RYR2*
rs16959717	16	58186000	C	A	0.010	5.44E-09	3.51E-01	*CNGB1*
rs12351692	9	100882441	A	G	0.010	8.30E-09	9.14E-01	*TRIM14*
rs2848646	2	158527128	C	T	0.015	1.27E-08	8.11E-01	*ACVR1*
rs12624187	2	34760954	A	G	0.028	1.66E-08	9.20E-01	*CRIM1*
rs7407765	18	76031697	A	C	0.026	2.09E-08	6.14E-01	*ATP9B*
rs6872783	5	34811080	A	G	0.039	2.25E-08	5.02E-01	*RAI14*
rs28823111	4	157645627	A	G	0.012	4.47E-08	3.80E-01	*PDGFC*
rs685352	15	35008335	G	A	0.45	4.61E-08	7.20E-09	*GJD2*

CHR: chromosome; BP: physical position of variant (genome build GRCh37; hg19); A1: effect allele; A2: non-effect allele; AF: allelic frequency of effect allele. Variants located outside genes (rs1917357, rs13375303, rs16959717, rs12624187, rs7407765, rs28823111, rs685352) were annotated to their nearest genes. Ordering according to the significance of each vQTL.
