# Supplementary material for: Gene-Environment Interaction Loci Associated with Refractive Error: SCAMPI Analysis
Source: Ophthalmol Sci. 2026 May 5;6(7):101219. doi: 10.1016/j.xops.2026.101219 (PMC13255065; doi:10.1016/j.xops.2026.101219)
Supplement: Supplementary Table 2 [file mmc3.pdf]

Supplementary Table 2. Gene-EduAge interactions associated with SER and AOSW for the 15 SCAMPI lead variants.

rsID	CHR	BP	A1	A2	AF	Annotation	Phenotype: SER			Phenotype: AOSW		
							BETA	SE	P	BETA	SE	P
rs12193446	6	129820038	G	A	0.096	*LAMA*	0.024	0.008	**3.82e-03**	0.17	0.033	**3.04e-07**
rs685352	15	35008335	G	A	0.45	*GJD2*	-0.015	0.005	**2.94e-03**	-0.096	0.020	**1.60e-06**
rs2117770	2	233375784	T	C	0.29	*PRSS56*	-8.11e-03	0.005	1.37e-01	-0.067	0.022	**2.16e-03**
rs7775087	6	73606783	G	T	0.44	*KCNQ5*	9.02e-02	0.005	7.21e-02	0.089	0.020	**8.10e-06**
rs13380104	15	79378821	T	C	0.42	*RASGRF1*	-0.010	0.005	**4.95e-02**	-0.071	0.020	**4.13e-04**
rs1405645	2	178853378	G	A	0.46	*PDE11A*	-5.93e-03	0.005	2.34e-01	-0.087	0.020	**1.34e-05**
rs869422	8	40723970	G	A	0.21	*ZMAT4*	0.015	0.006	**1.61e-02**	0.084	0.024	**6.13e-04**
rs11079249	17	54716686	A	G	0.36	*NOG*	5.68e-03	0.005	2.72e-01	0.023	0.021	2.63e-01
rs2969230	17	11419528	C	T	0.48	*SHISA6*	6.09e-03	0.0049	2.18e-01	-0.044	0.020	**2.47e-02**
rs7903931	10	79114690	C	T	0.36	*KCNMA1*	-6.57e-03	0.005	2.00e-01	-0.053	0.021	**1.06e-02**
rs10113215	8	60132194	G	A	0.33	*TOX*	6.75e-03	0.005	1.98e-01	0.092	0.021	**1.24e-05**
rs10509491	10	85977175	A	G	0.47	*CDHR1*	6.65e-03	0.005	1.79e-01	0.019	0.020	3.32e-01
rs7077247	10	114812071	C	T	0.46	*TCF7L2*	-0.013	0.005	**7.45e-03**	-0.026	0.020	1.98e-01
rs4794029	17	47280301	T	C	0.32	*GNGT2*	0.011	0.005	**3.62e-02**	0.050	0.021	**2.03e-02**
rs2229741	21	16340289	T	C	0.42	*ASMER1*	1.11e-03	0.005	8.24e-01	-0.038	0.020	5.56e-02

SER: spherical equivalent refraction; AOSW: age of onset of spectacle wear; CHR: chromosome; BP: physical position of variant (genome build GRCh37; hg19); A1: effect allele; A2: non-effect allele; AF: allelic frequency of effect allele. Variants located outside genes (rs685352, rs2117770, rs11079249, rs10113215) were annotated to their nearest genes. vQTL: variance quantitative locus; EduAge: age completed full-time education; BETA: effect size; SE: standard error. Bold P values highlighted vQTLs which were at least nominally significant ($P<0.05$). Ordering according to the significance of each vQTL in SCAMPI analysis.
