# Supplementary material for: Gene-Environment Interaction Loci Associated with Refractive Error: SCAMPI Analysis
Source: Ophthalmol Sci. 2026 May 5;6(7):101219. doi: 10.1016/j.xops.2026.101219 (PMC13255065; doi:10.1016/j.xops.2026.101219)
Supplement: Supplementary Table 4 [file mmc4.pdf]

Supplementary Table 4. Gene-age interactions associated with SER and AOSW for the 15 SCAMPI lead variants.

rsID	CHR	BP	A1	A2	AF	Annotation	Phenotype: SER			Phenotype: AOSW		
							BETA	SE	P	BETA	SE	P
rs12193446	6	129820038	G	A	0.096	*LAMA*	-5.91e-04	0.003	8.25e-01	-0.030	0.011	**7.90e-03**
rs685352	15	35008335	G	A	0.45	*GJD2*	0.001	0.002	4.37e-01	0.014	0.007	**4.30e-02**
rs2117770	2	233375784	T	C	0.29	*PRSS56*	0.002	0.002	1.63e-01	0.005	0.007	5.35e-01
rs7775087	6	73606783	G	T	0.44	*KCNQ5*	0.002	0.002	1.96e-01	-0.019	0.007	**4.94e-03**
rs13380104	15	79378821	T	C	0.42	*RASGRF1*	1.77e-04	0.002	9.11e-01	0.015	0.007	**2.78e-02**
rs1405645	2	178853378	G	A	0.46	*PDE11A*	-2.44e-04	0.002	8.78e-01	0.010	0.007	1.26e-01
rs869422	8	40723970	G	A	0.21	*ZMAT4*	9.98e-04	0.002	6.11e-01	-0.002	0.008	7.99e-01
rs11079249	17	54716686	A	G	0.36	*NOG*	0.001	0.002	4.10e-01	-0.010	0.007	1.59e-01
rs2969230	17	11419528	C	T	0.48	*SHISA6*	-8.00e-04	0.002	6.12e-01	0.004	0.007	5.64e-01
rs7903931	10	79114690	C	T	0.36	*KCNMA1*	-6.34e-04	0.002	6.99e-01	0.005	0.007	4.46e-01
rs10113215	8	60132194	G	A	0.33	*TOX*	0.001	0.002	4.45e-01	0.002	0.007	7.80e-01
rs10509491	10	85977175	A	G	0.47	*CDHR1*	0.002	0.002	2.48e-01	-0.007	0.007	2.97e-01
rs7077247	10	114812071	C	T	0.46	*TCF7L2*	-0.001	0.002	4.63e-01	-0.004	0.007	5.57e-01
rs4794029	17	47280301	T	C	0.32	*GNGT2*	0.002	0.002	1.53e-01	-0.009	0.007	1.96e-01
rs2229741	21	16340289	T	C	0.42	*ASMER1*	-0.001	0.002	4.09e-01	5.43e-06	0.007	9.99e-01

SER: spherical equivalent refraction; AOSW: age of onset of spectacle wear; CHR: chromosome; BP: physical position of variant (genome build GRCh37; hg19); A1: effect allele; A2: non-effect allele; AF: allelic frequency of effect allele. Variants located outside genes (rs685352, rs2117770, rs11079249, rs10113215) were annotated to their nearest genes; BETA: effect size; SE: standard error. Bold P values highlighted vQTLs which were at least nominally significant ($P<0.05$). Ordering according to the significance of each vQTL in SCAMPI analysis.
