# Supplementary material for: Gene-Environment Interaction Loci Associated with Refractive Error: SCAMPI Analysis
Source: Ophthalmol Sci. 2026 May 5;6(7):101219. doi: 10.1016/j.xops.2026.101219 (PMC13255065; doi:10.1016/j.xops.2026.101219)
Supplement: Supplementary Table 5 [file mmc5.pdf]

Supplementary Table 5. Non-additive effects associated with SER and AOSW for the 15 SCAMPI lead variants.

rsID	CHR	BP	A1	A2	AF	Annotation	Phenotype: SER			Phenotype: AOSW		
							BETA	SE	P	BETA	SE	P
rs12193446	6	129820038	G	A	0.096	*LAMA*	0.120	0.051	**1.91e-02**	0.210	0.20	3.06-01
rs685352	15	35008335	G	A	0.45	*GJD2*	0.049	0.018	**6.37e-03**	0.100	0.07	1.71e-01
rs2117770	2	233375784	T	C	0.29	*PRSS56*	0.050	0.021	**2.09e-02**	0.120	0.09	1.53e-01
rs7775087	6	73606783	G	T	0.44	*KCNQ5*	0.037	0.018	**4.08e-02**	0.100	0.07	1.57e-01
rs13380104	15	79378821	T	C	0.42	*RASGRF1*	-0.008	0.018	6.73e-01	0.063	0.07	3.83e-01
rs1405645	2	178853378	G	A	0.46	*PDE11A*	-0.011	0.018	5.46e-01	-0.039	0.07	5.82e-01
rs869422	8	40723970	G	A	0.21	*ZMAT4*	0.130	0.027	**7.99e-07**	0.310	0.11	**4.89e-03**
rs11079249	17	54716686	A	G	0.36	*NOG*	-0.014	0.019	4.51e-01	0.100	0.08	1.90e-01
rs2969230	17	11419528	C	T	0.48	*SHISA6*	-0.041	0.018	**1.93e-02**	-0.120	0.07	7.94e-02
rs7903931	10	79114690	C	T	0.36	*KCNMA1*	0.026	0.019	1.72e-01	0.067	0.08	3.83e-01
rs10113215	8	60132194	G	A	0.33	*TOX*	0.020	0.020	3.20e-01	0.042	0.08	5.99e-01
rs10509491	10	85977175	A	G	0.47	*CDHR1*	-0.032	0.018	7.10e-02	-0.035	0.07	6.24e-01
rs7077247	10	114812071	C	T	0.46	*TCF7L2*	0.005	0.018	7.90e-01	0.021	0.07	7.69e-01
rs4794029	17	47280301	T	C	0.32	*GNGT2*	0.029	0.021	1.53e-01	0.100	0.08	2.08-01
rs2229741	21	16340289	T	C	0.42	*ASMER1*	0.015	0.018	3.97e-01	-0.098	0.07	1.78e-01

SER: spherical equivalent refraction; AOSW: age of onset of spectacle wear; CHR: chromosome; BP: physical position of variant (genome build GRCh37; hg19); A1: effect allele; A2: non-effect allele; AF: allelic frequency of effect allele. Variants located outside genes (rs685352, rs2117770, rs11079249, rs10113215) were annotated to their nearest genes. BETA: effect size; SE: standard error. Bold P values highlighted vQTLs which were at least nominally significant ($P<0.05$). Ordering according to the significance of each vQTL in SCAMPI analysis.
