# Supplementary material for: Gene-Environment Interaction Loci Associated with Refractive Error: SCAMPI Analysis
Source: Ophthalmol Sci. 2026 May 5;6(7):101219. doi: 10.1016/j.xops.2026.101219 (PMC13255065; doi:10.1016/j.xops.2026.101219)

Supplementary Figure. Q-Q plot of SCAMPI, Levene's test and CQR. Systematic inflation of p-values was observed for all methods: λ_{GC} = 1.18 for SCAMPI (A), λ_{GC} = 1.10 for Levene's test (B) and λ_{GC} = 1.13 for CQR (C). Genomic control was used to account for this issue. Results without corrections are denoted as 'raw'; results after correction are denoted as 'adjust'.

The black solid line represented the observed –log10(p-values) vs. expected –log10(p-values) under the null hypothesis.

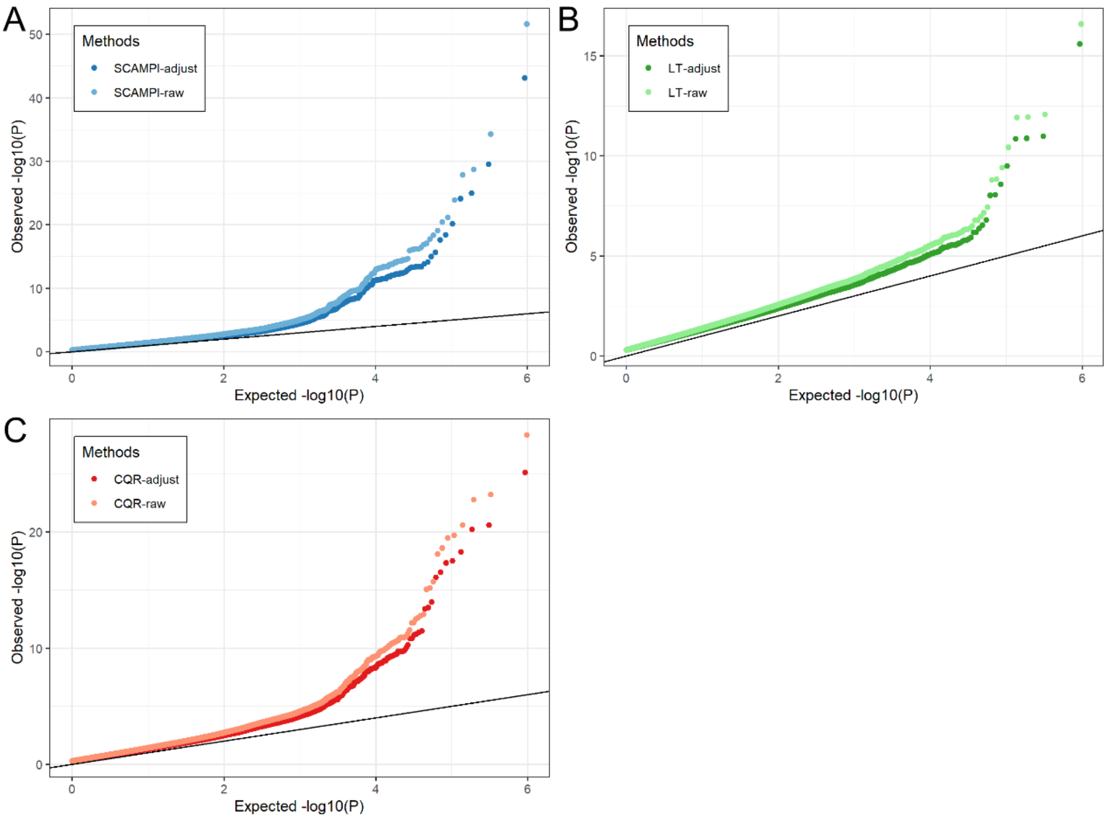

Supplement: Supplementary Figure [file mmc6.pdf]
